# Aging and stress induced β cell senescence and its implication in diabetes development

**DOI:** 10.18632/aging.102432

**Published:** 2019-11-13

**Authors:** Na Li, Furong Liu, Ping Yang, Fei Xiong, Qilin Yu, Jinxiu Li, Zhiguang Zhou, Shu Zhang, Cong-Yi Wang

**Affiliations:** 1 The Center for Biomedical Research, Key Laboratory of Organ Transplantation, Ministry of Education, NHC Key Laboratory of Organ Transplantation, Key Laboratory of Organ Transplantation, Chinese Academy of Medical Sciences, Tongji Hospital, Tongji Medical College, Huazhong University of Science and Technology, Wuhan, China; 2 Department of Dermatology, The People’s Hospital of Shishou City, Shishou, Hubei, China; 3 Shenzhen Third People’s Hospital, Longgang District, Shenzhen, Guangdong, China; 4 Diabetes Center, The Second Xiangya Hospital, Institute of Metabolism and Endocrinology, Central South University, Changsha, China

**Keywords:** senescence, β cell, β cell regeneration, insulin secretion, diabetes

## Abstract

Cellular senescence is a well-established defensive mechanism for tumor suppression, and is also proposed to play a crucial role in embryonic development, wound repair, aging and age-related diseases. Senescent cell is characterized by the marked morphological changes and active metabolism along with a distinctive senescence associated secretion phenotype (SASP). Cellular senescence is triggered by multiple endogenous and exogenous stressors, which collectively induce three types of senescence. It is believed that senescence represents a programmed phenomenon to facilitate β cell functional maturation and, therefore, senescence has been suggested to be involved in β cell regeneration, insulin secretion and diabetes development. Nevertheless, despite past extensive studies, the exact impact of senescence on β cell viability, regeneration and functionality, and its relevance to the development of diabetes are yet to be fully addressed. In this review, we will summarize the recent progress in β cell senescence, through which we intend to spark more instructive discussion and perspective with regard to the mechanisms underlying β cell senescence and their links to the pathogenesis of diabetes and the development of therapeutic strategies.

## INTRODUCTION

Cellular senescence is defined as the irreversible arrest of cell proliferation. In the 1960s, Hayflick et al. found that normal human diploid fibroblasts entered into an irreversible non-dividing state after a certain number of divisions, which was referred to as “Hayflick limit” [1]. Since then, multiple types of cellular senescence have been identified including replicative senescence, oncogene-induced senescence, DNA damage-induced senescence, oxidative stress-induced senescence, chemotherapy-induced senescence, mitochondrial dysfunction-associated senescence, epigenetically induced senescence, paracrine senescence, wound healing and embryonic development related senescence [2]. However, whether all of those types of senescence model occur *in vivo* still remains unknown.

In general, senescent cells are characterized by the enlarged cell size, increased lysosomal content and upregulated β-galactosidase activity at nearly pH 7.0 [3]. Cellular senescence is established and maintained by at least two major tumor suppressor pathways [4], the p53/p21 and the p16^Ink4a^/retinoblastoma protein (Rb) axes. It is believed that the p53/p21 axis initiates the senescence process, while the p16^Ink4a^ activation maintains the senescence state [5]. In cultured cells, senescence occurs as a defensive mechanism to resolve cellular insults, leading to transient cell cycle arrest. In this case, cells can re-enter cell cycle once the stress is resolved. Prolonged cellular stress (> 4 days), however, spurs permanent senescence [6]. Other than ceased cell division, senescent cells also display widespread changes in chromatin structure (referred to as senescence associated heterochromatin foci, SAHF) and gene expression profiles [7], which synergistically lead to highly active cellular metabolism and massive secretion of cytokines (TGF-β, IL-1a, -1β and -6), chemokines (IL-8, CXCL1), growth factors (FGF, HGF) and proteases (MMP-1, -3, and -13), collectively defined as senescence associated secretory phenotypes (SASP) [3, 8]. Interestingly, senescent cells manifest loss of Lamin B1 expression, but the related mechanism and significance are yet to be explored [9]. SASP is a characteristic feature shared by almost all senescent cells, and it is mainly initiated by the NF-κB and p38MAPK pathways, while maintained by IL-1α in an autocrine manner [10]. The composition of the senescence-associated secretome varies depending on the time spent in senescence, the senescence inducer and the cell type [11]. Two main distinct secretomes have been described, and the NOTCH1 signaling plays a pivotal role in switching secretome composition [12]. During the early stage of senescence, NOTCH1 activity fluctuates dynamically, which triggers a TGF-β rich secretome to suppresses the senescence-associated pro-inflammatory secretome by inhibiting C/EBPβ signaling. However, sustained senescence endows NOTCH1-driven TGF-β to repress NOTCH1 signaling transduction, which in turn contributes to the second wave of senescence induction, thereby changing the TGF-β rich secretome into a pro-inflammatory-centered one [12, 13].

It is believed that senescence represents a programmed phenomenon that facilitates mammalian embryonic development and β cell functional maturation after birth [14, 15]. A couple of senescence hallmarks including p16^Ink4a^, p19^Arf^ and p15^Ink4b^ increase in pancreatic β cells during aging, along with decreased capability of regeneration [16–18]. A large body of work has focused on the impact of senescent β cell accumulation on the pathogenesis of type 1 diabetes (T1D) and progression of age-related type 2 diabetes (T2D) [19, 20]. These studies open up new perspectives to understand aging and diabetes development, which would promote the exploitation of promising therapeutic strategies. In this review, we summarize the recent progress in aging-, and stress-induced β cell senescence, and its impact on β cell viability, insulin secretion and regeneration, as well as discuss its relevance to the development of diabetes mellitus.

## Characteristics of β cell senescence

Studies in rodents and humans have revealed that recovery and plasticity of islet cells decrease in mice once they reached 1-year of age, and human β cell population is established by the age of 20 [21] except for the existence of a small population of “virgin β cell”, which is functionally immature [22]. Those facts are reminiscent of natural β cell senescence with age. Generally, senescent β cells exhibit larger cell size (~14um) than the normal (~12um), and can be featured by the upregulated expression of *Cdkn2a/1a* (encoding p16^Ink4a^ and p21, respectively) and anti-apoptotic molecules (e.g., Bcl-2, -xl and -w) along with senescence associated β-galactosidase staining [19, 20]. Specific composition of senescence-associated secretome helps to distinguish β cell senescence with senescence in other cell types. Of note, distinctive features and signatures of β cell senescence exist in T1D and T2D disease models in multiple ways, indicating that β cell senescence is dynamically regulated under different cellular contexts.

Aging and stress (e.g., hyperglycemia, viral insult, inflammatory response and insulin resistance) can be contributors to β cell senescence. Studies have demonstrated that aging causes massive changes of β cell chromatin accessibility, leading to significant alterations in gene expression profile [23]. Nevertheless, the association of senescence phenotype with β cell epigenetic and transcriptomic changes during aging still need further exploration. Indeed, single cell RNA-seq analysis demonstrated that aged human pancreas manifests enhanced transcriptional noise, somatic mutations and senescence signatures [24]. Emerging evidence shows that sources and levels of DNA damage increase with age along with decreased DNA repair capacity [25], predisposing β cells to cell cycle arrest and DNA damage response (DDR) associated with senescence. Cell replication-related telomere erosion is known to directly associate with lifespan limitation [26]. There are data supporting that short telomere impairs β cell function and participates in β cell destruction in the late stage of T2D [27]. Furthermore, proteomic analysis reveals that β cells manifest a significant discrepancy in terms of the expression of aging markers (e.g., IGF1R) between islets even within the same islets, suggesting that β cells display a remarkable aging heterogeneity [28]. Hyperglycemia is another trigger of β cell senescence. β cells maintain blood glucose homeostasis by controlling appropriate insulin secretion according to the real-time changes of blood glucose levels [29]. Sustained hyperglycemia, however, would induce β cell senescence *via* multiple possible mechanisms, such as apoptosis signaling-regulating kinase 1 activation [30], p38 mitogen-activated protein kinase activation [31], and “glycolytic overload” (characterized by the increased metabolic flux through glycolysis in hyperglycemia and the decreased proteolysis of hexokinase) mediated mitochondrial dysfunction [32]. Importantly, unlike other cell types, β cells manifest relatively lower antioxidant capability and, as a result, they are more susceptible to oxidative stress and endoplasmic reticulum (ER) stress [33–35]. Excessive reactive oxygen species (ROS) production impairs mitochondrial dynamics (fission and fusion), leading to defective electron transport chain, bioenergetics imbalance, and altered mitochondria calcium homeostasis, which then trigger β cell senescence [36–38]. It is noteworthy that mitochondria-related senescence is featured by the lack of IL1-arm cytokines, due to high AMP to ATP ratio in the mitochondria coupled with highly activated AMPK, which in turn represses the initiation of mTORC1 and IL1-arm cytokine responses [39]. This feature, however, has not been validated in β cells.

Increased protein synthesis load, oxidative stress, gene mutations, glucolipotoxicity, can cause ER tress in β cells. The activation of the three branches of unfolded protein response accelerates cellular senescence in non-β cell types, and the ER chaperone, Bip, has a possible central role in senescence [38, 40]. Therefore, it is quite possible that ER stress actively participates in β cell senescence despite the unclear molecular mechanisms. Virus, especially enteroviruses, is one of the origins of β cell DNA damage [41]. Indeed, recent studies demonstrated that islets with infiltrated immune cells are characterized by the increased frequency of DDR and enhanced expression of senescence markers in newly diagnosed T1D patients and rodent T1D model [42], indicating that DNA damage-induced β cell senescence may play a critical role in the early stage of autoimmune diabetes. Up to now, however, the contribution of virus insult to β cell senescence and T1D progression remains to be described. Nevertheless, the existing data support that autoimmune response in T1D and chronic inflammation in T2D are suspect culprits of β cell senescence, possibly through ER stress, DNA damage and other signaling pathways. While systemic insulin resistance accelerates β cell senescence during aging, the involved molecular mechanisms, however, are not fully understood. Collectively, during the course of aging and diabetes progression, multiple triggers and signaling pathways collaborate and twist together to induce β cell senescence, leading to changes in β cell functions and systemic metabolic homeostasis in cell-autonomous and noncell-autonomous manners.

## The effect of senescence on β cell regeneration

β cell cycling is driven by CyclinD1/2-CDK4 activity and downregulated by CDK inhibitor p16^Ink4a^. It has proven that β cell expansion is an age-dependent process, and β cell replication is much more robust in young mice than that in old animals [43]. Once p16^Ink4a^ is specifically expressed, pancreatic β cells show obvious senescence phenotype along with compromised cell regeneration [17]. Since p16^Ink4a^ transcript is enriched in purified islets when compared with exocrine tissues, supporting that p16^Ink4a^ serves as a crucial checkpoint in β cell senescence as well as proliferation. Conversely, p16^Ink4a^ ablation enhances β cell proliferation, especially in the case of β cells following toxic insult [16]. It has been shown that two chromatin-regulating polycomb group proteins, Bmi1 and Ezh2, are related to age dependent high levels of p16^Ink4a^ expression in β cells, suggesting that epigenetic regulation could be involved in senescence-mediated aging and type 2 diabetes [44, 45]. Indeed, mice deficient in pituitary tumor transforming gene (PTTG), which encodes a securing protein that regulates chromosome separation, go through evident senescence and apoptosis in islet β cells at 2-month-old [46]. Enhanced p21 expression in β cells following PTTG knockout could be one of the contributors to senescent phenotype, because p21 deletion only partially rescued mice from diabetes resulted from severe β cell diminishment. Those discoveries support the notion that β cell senescence can be secondary to DNA damage associated gene activation, and furthermore, additional PTTG downstream genes may also synergize with p21 attributing to β cell senescence and cycling arrest.

**Figure 1:**
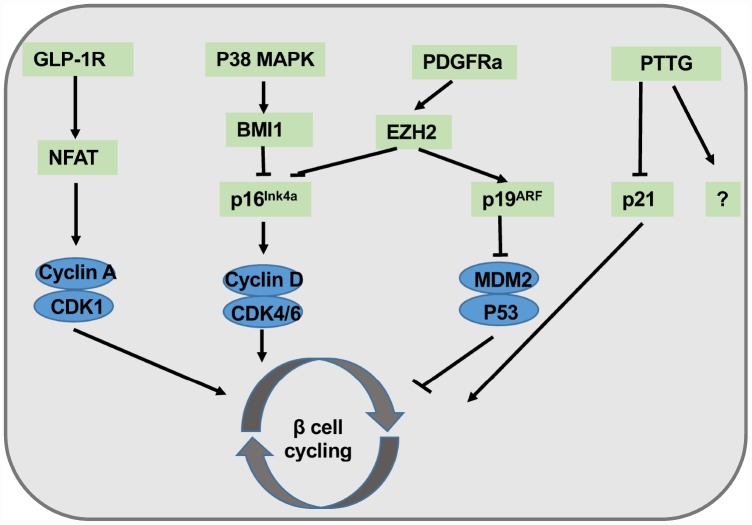
**Summary of molecular pathways involved in β cell regeneration.** Exendin-4 agonizes GLP-1R signaling, followed by the activation of NFAT and the entry of cell cycling. P38 MAPK signals activate BMI1 and inhibit p16^Ink4a^. PDGFRa transduces proliferative signals to EZH2, thereby attenuating p16^Ink4a^ activity while enhancing p19^Arf^ activation. PTTG partially promotes β cell proliferation *via* p21 inhibition.

To develop the strategies for β cell expansion against aging, a great deal of research effort has been focused on the regulation of β cell regeneration/senescence. Platelet-derived growth factor (PDGFR) signaling has been characterized to play a critical role in cellular proliferation and development. PDGFR losses its expression with age both in mouse and human islet β cells along with deceased EZH2 expression [45]. Conditional over-activation of PDGFRa in β cells enhances neonatal β cell propagation and regeneration in adult islets [47]. Juvenile human islets rather than adult islets exposed to PDGF-AA, a PDGF-A agonist, rejuvenate β cell proliferation. Similarly, exendin-4, an agonist of the glucagon like peptide 1 receptor (GLP-1R), successfully stimulates juvenile human β cell expansion but fails in adult islet β cells [48]. These findings are expected, because aging process causes overall changes of chromatin accessibility and gene expression profile toward the activation of metabolic regulator and suppression of proliferation program, which is a reminiscent of irreversibility of normal aging (Figure 1).

## The influences of β cell senescence in insulin secretion

The implication of aging in the regulation of insulin synthesis/secretion and glucose homeostasis had been recognized back to 1980s. By utilizing Fischer rat, an established aging animal model, Wang etal. found that aging has no effect on preproinsulin mRNA transcription, but impairs nearly half amount of proinsulin synthesis upon high glucose stimulation [49]. Undoubtedly, decreased proinsulin synthesis would lead to the reduction of newly formed insulin secretion. Given that pancreatic weight, total insulin content, islet size and mean insulin content per islet are unchanged, the impairments in the signal transduction following glucose stimulation during aging process could be a crucial culprit. Indeed, studies with time-dependent potentiation (TDP) of insulin release in aged rats confirmed that β cells lose sensitivity to secretagogues during aging process [50]. However, the exact mechanisms had not been dissected at that time. Actually, in a β cell specific p16^Ink4a^ overexpression mouse model, ectopic p16^Ink4a^ expression improved glucose stimulated insulin secretion response apart from cell cycle arresting [17]. Although this finding is consistent with previous data demonstrating that mitochondrial metabolism and insulin exocytosis relevant to β cell functions are improved during aging process, but contradicts to other findings [23, 49, 50]. The following possible reasons may explain the above discrepancies. On one hand, transgenic p16^Ink4a^ expression in β cell may only simulate one facet of cellular senescence to partially reflect β cell senescence phenotype, but sustained decrease of β cell function in elderly individuals cannot be neglected [50]. On the other hand, insulin synthesis and secretion in aged subjects are likely modulated by multiple factors such as senescence marker protein-30 (SMP-30), an androgen independent factor involving in Vitamin C synthesis that decreases during aging process to impair GSIS in elders [51]. Furthermore, elevated plasma level of deoxysphingolipid is responsible for the senescent characteristics and compromised GSIS both in INS-1 cells and primary islets [52]. Another noteworthy phenomenon is that senescent β cells manifest higher basal insulin level (2.8mM glucose), which is similar to the immature β cell phenotype. This puzzle may be partially explained by NAD(P)H fluorescence lifetime imaging (FLIM) implication. Aging causes β cell mitochondria dysfunction mainly through complex I/II disorder followed by reduction of KATP channel activity and increase of Ca2^+^ inflowing that occur as a compensatory strategy, thereby increasing insulin exocytosis [53].

**Figure 2:**
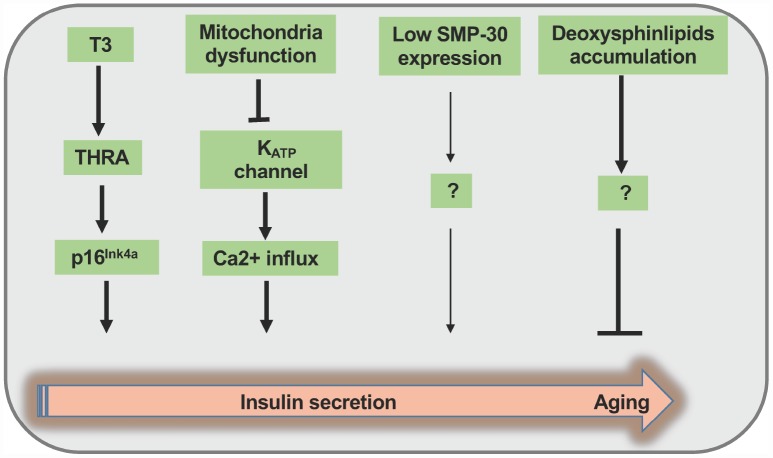
**Summary of the regulation of insulin secretion during the course of aging process.** Thyroid hormone (T3) promotes β cell functional maturation *via* the induction of MafA expression along with p16^Ink4a^ activation at the early stage of aging. During the progression of aging process, impaired mitochondria function causes KATP channel shutting down and Ca2^+^ influx at the same time, thereby enhancing insulin secretion in a short time frame. In contrast, during the advanced aging process, β cells manifest diminished expression of senescence marker protein-30 (SMP-30) along with deoxysphingolipid accumulation, thereby impeding insulin secretion through unknown mechanisms.

More recently, studies indicated that β cell senescence can be affected by proximal pancreatic cells namely acinar cells and other hormonal factors. It was noted that the expression of arginase II in acinar cells increases during aging process, and enhanced TNF-α release from acinar cells induces β cell dysfunction and apoptosis [38]. Since β cell senescence can be disseminated by surrounding β cells, a crosstalk may exist between β cells and other types of pancreatic cells, thereby regulating β cell senescence. Indeed, thyroid hormone (T3) spurs β cell functional maturation through MafA induction, and enhances cell senescence by directly activating its target p16^Ink4a^ [54] through TH receptor B (THRB) and TH receptor A (THRA). Arum et al. demonstrated that mice deficient in growth hormone receptor (GHR)/binding protein gene display hypoinsulinemia, higher sensitivity to insulin and prolonged lifespan even though they are smaller in size [55], while insertion of *Igf1* gene under the control of a rat insulin promoter (RIP) can reverse the above phenotypes, supporting that IGF1R is a new aging marker [28, 55]. Consistently, transgenic *Igf1* expression in conventional GHR knockout mice highlights that insulin sensitivity is important for longevity, and the pace of aging can actually be hormonally regulated [56]. (Figure 2)

It would be necessary to keep in mind that several essential questions need to be fully addressed to dissect the association between senescence and β cell function thoroughly. First, how does senescence regulate proinsulin synthesis in β cells? second, how many glucose responding proteins are subject to β cell senescence? and third, how does the enhanced basal insulin secretion occur and what is the relevance to aging or diabetes mellitus?

## The relevance of β cell senescence to diabetes development

T1D is featured by the progressive β cell destruction from autoimmune response. Recently, Thompson et al. demonstrated that a subpopulation of senescent β cells exists in both NOD mice and human T1D patients, and they actively recruit autoimmune cells, indicating a pivotal role of β cell senescence in T1D progression [20]. Specifically, in T1D-prone NOD mice, the autoimmune cells initiate peri-insulitis (the recruitment of autoreactive immune cells into the periphery of islets) with minor β cell destruction from 3-4 weeks to 8-10 weeks, while the disease progresses after 10 weeks, which causes invasive insulitis accompanied by the massive β cell destruction and severe hyperglycemia [57]. The single β cell transcription profile comparison between the two stages of T1D reveals that senescent β cells accumulate with disease progression, exhibit DNA damage and stress-induced senescence phenotypes. The senescent cells are conspicuous due to the upregulated senescent markers consist of p16^Ink4a^, p21, Ser139-phosphorylated histone H2A.X (γ-H2A.X), the increased SASP markers including *Cxcl10*, IL-6, Mmp-2 and Flnb, as well as specific secretome (including IL-6, Igfbp3 and Serpine1). Notably, the SASP factors potently enforce the paracrine effect of senescence and the chemotaxis of immune cells. When translating those findings to human T1D cases, senescent human β cells display some distinctive characteristics. Firstly, in human β cells, p16^Ink4a^ is more likely to be an age-related senescence marker rather a T1D-related one. In contrast, p21 expression drastically increases in autoantibody-positive nondiabetic donors and newly diagnosed T1D donors, which renders p21 to be a human T1D-related senescence marker. Secondly, the senescent secretome in human T1D is featured by IL-6 and Serpine1 expression. Lastly, heterogeneity among islets and individuals is obvious in terms of the aforementioned senescent marker expression levels. The senescent NOD β cells highly express Bcl-2, but the anti-apoptotic feature in human senescent β cells, however, remains undefined due to unknown limitations. While human T1D onset can take a couple of years or decades, stress-induced, instead of age-related senescence, take the primary responsibility for disease progression. There is strong evidence that autonomous β cell DDR along with the presence of the hallmarks of senescence can play a causal role in autoimmune initiation and progression during the course of T1D development [42]. However, β cell DNA damage caused by autoimmunity does not involve in this process as the major senescent β cell in NOD islets are not surrounded by immune cells.

T2D is an aging-associated disease characterized by the systemic insulin resistance and metabolic dysfunction in multiple organs and tissues, which includes two stages, the pre-diabetes stage and the early-stage diabetes. The pre-diabetes stage can sustain for many years attributing to β cell compensation (i.e., increased β cell mass and workload) till β cell compensation failure, which leads to the second stage featured by β cell death, decreased insulin levels in the circulation and prolonged hyperglycemia [58]. The boundary of the two stages, however, is difficult to define since most patients remain in a grey area of diagnosis, where diet changes and antidiabetic drugs are sufficient to maintain normoglycemia. The connection between β cell senescence and T2D has been established for several years [59]. To understand the effects of β cell senescence on T2D pathogenesis, aging and stress factors including hyperglycemia, hyperlipidemia and chronic inflammation, should be taken into account. Aged β cells accumulate with age, which exhibit impaired insulin secretion response to glucose challenges. It was noted that β cells in aged C57BL/6 mice display a distinctive transcription profile characterized by the downregulation of β cell identity genes (including *Insulin1*, *MafA*, *Nkx6.1*, and *Pdx1*), upregulation of senescence markers (including *Cav1*, *Cdkn1a*, *Id2*, *Makp2k1*, *Prkcd*, *Tbx2* and *Tbx3*), SASP genes (e.g., *Ccl2*, *Cd68*, *Igfbp3*, *Il6*, *Tnf* and *Serpine1*) and disallowed genes that are supposed to be repressed in β cells, such as *Ldha* and *Catalase* [19, 60]. Nevertheless, those mouse-derived data are only partially consistent with what found in aged human islets [28]. It is noteworthy that insulin resistance can exacerbate the senescent process in aged β cells [19]. However, unlike high-fat-diet induced senescence, β cell senescence induced by acute administration of S961, an insulin receptor antagonist, can be reversed once S961 is withdrawal [61]. Despite the unclear mechanisms, this finding indicates that β cell senescence can be reversed at early stage, and therefore, the impact of β cell senescence on clinical T2D progression could be much more complicated than what we thought. Indeed, in humans, the proportion of senescent β cells (characterized by the upregulation of *CXCL10*, *CCL4,* *IL1A* and *IL6*) substantially increased in aged subjects, and further increased in subjects with T2D. However, only two SASP factors (CCL4 and IL6) are detected in human T2D patients, left the impact of β cell senescence on human T2D progression an unfinished story [62]. Nevertheless, insulin resistance has been consistently found to be tightly associated with hyperglycemia, hyperlipidemia and chronic inflammation both in T2D patients and animals, and those factors are linked to the occurrence of β cell senescent phenotypes and SASP activities. In the pre-diabetes stage, insulin-resistance-related high blood glucose, dyslipidemia and inflammation increase insulin demand, impelling the β cell expansion to secret more insulin [14]. β cell adaptive regeneration shortens telomere and activates DDR, followed by cellular senescence. On the other hand, paracrine senescence accelerates senescent β cell accumulation and promotes SASP activities, which in turn exacerbate systemic insulin resistance and enhance the loss of β cell compensation, coupled with the progression of pre-diabetes to the early diabetes stage. However, it would be difficult to define in which diabetes stage that β cell senescence is more important, because of the vague boundary of the two stages and the prolonged timeframe of β cell senescence.

Recently, Dooley et al. identified β cell variations in genes *Xrcc4* and *Glis3* in the NOD islets, both of which share links to T2D susceptibility [63, 64], supporting genetic predisposition of β cell senescence in diabetes risk [65]. Furthermore, SNPs adjacent to the *CDKN2a/b* gene have been identified and attested to associate with T2D in large GWAS studies [66]. These discoveries highlight that β cell senescence related genetic defects may increase the susceptibility of T1D and T2D. Collectively, β cell senescence is a common contributor to T1D and aging-related T2D. It seems that DDR is a common trigger of β cell senescence both in T1D and aging-associated T2D. However, whether this pathway is the most essential one to drive β cell senescence and diabetes onset remains to be further explored. Obviously, distinctive origins of β cell senescence delineate different senescence signatures and secretomes, suggesting distinctive senescent mechanisms and SASP effects on T1D and T2D. Since cellular senescence is a dynamic process varying with cell type, senescence inducer and time of duration, those properties in β cell senescence, however, have not been fully described in present studies.

## Concluding remarks and future directions

Cellular senescence is certainly crucial to growth and development at early stage of life. Specifically, senescence promotes β cell functional maturation including increased glucose uptake, mitochondrial oxidation capability and mitochondria number. However, sustained senescence is associated with aging-related lifespan limitation and disease development. β cell senescence during aging impairs the expression of genes relevant to β cell identity and cellular functions. Furthermore, SASP-derived cytokines could trigger inflammatory response, which renders β cell status even worse. Although the established mouse model with p16^Ink4a^ overexpression in around 35% of β cells largely resembles the phenotypes observed in normal aging mice, but some discrepancies are noted. For example, aged mice show impaired response to glucose fluctuations, whereas p16^Ink4a^ overexpression merely improves high glucose stimulated insulin secretion. Therefore, cellular senescence is a more complicated entity involving multiple molecules and signaling pathways [9]. As such, p16^Ink4a^ overexpressing cells just manifest a part of senescence phenotype, but lack of other upstream/downstream signals and cellular alterations. Particularly, aged human islets display an age dependent decline in the coordination of Ca^2+^ dynamics, gap junction coupling and insulin secretion [67], and p16^Ink4a^ mediated improvement of GSIS does not seem to be durable, because deteriorated glucose tolerance is noted once induced p16^Ink4a^ expression lasts for 5-month.

Studies in a genetic senescence activation mouse model revealed that prolonged β cell senescence deteriorates cellular function followed by β cell exhaustion and β cell death no matter what type of cell death it is [17]. Furthermore, during the course of natural aging process, islet cells from both aged human and rat are sensitive to glucose induced β cell apoptosis confirmed by TUNEL staining [68]. Given that senescent cells are resistant to apoptosis, the effect of senescent cells is mostly dependent on their SASP activities including paracrine senescence and chemotaxis, which may explain β cell destruction and decreased β cell mass associated hyperglycemia. A critical question is whether targeted clearance of senescent cells would attenuate diabetes development. To address this question, large number of pharmacological compounds (defined as “senolytics”) have been identified to specifically induce senescent cell death [2]. Those senolytic drugs are aimed at combating some chronic diseases (e.g., diabetes, neurodegenerative diseases) or extending lifespan, even though with possible unclear side effects [69–74]. In this case, the effect of various senolytic chemicals have been exploited intensively on multiple disease models including T1D and T2D [19, 20, 75]. Specifically, senolytic induction of senescent β cells by two BH3 mimetics, ABT-737 (inhibits Bcl-2, Bcl-xl and Bcl-w) [76] and ABT-199 (a FDA-approved drug which inhibits Bcl-2 specifically) [77], effectively halts T1D development in NOD mice [20]. Similarly, ABT-263 reverses T2D outcomes by improving β cell function and identity [19, 78]. In consideration of the pros and cons of these senolytic drugs on trial, next generations of potent senolytic compounds targeting diabetes with minor side effects should be developed urgently based on β cell senescence and SASP properties. Encouragingly, several approved antidiabetic drugs actually exhibit anti-senescence effect to some extents. For example, metformin has now been tested in the Targeting Aging with Metformin trials as a good regimen against aging and age-related diseases [79–82]. Since calorie restriction improves longevity [83, 84], and metformin treatment just mimics dietary restriction, which highly upregulates AMPK activity while inhibits mTORC1 activity and NF-κB pathway, thereby modulating autophagy/senescence and ameliorating cardiovascular diseases [85, 86]. It has been noted that chronic low dose metformin treatment extends lifespan by increasing the nuclear accumulation of nuclear factor erythroid 2-related factor 2 (Nrf2) to facilitate an array of expressions for antioxidant genes [60]. Additionally, dipeptidyl-peptidase 4 inhibitor and rosiglitazone also display plausible effects on ameliorating senescence in non-β cell types [87], unveiling novel molecular mechanisms in antidiabetic therapies.

At present, several questions are still remained to be investigated in terms of the role of β cell senescence in diabetes pathogenesis. First, as β cell senescence has been associated with both T1D and T2D, detailed mechanistic studies are necessary to explain how β cell senescence affects diabetes development (e.g., the role of β cell senescence in the initiation and progression of diabetes). Second, previous studies suggested links between senescence and autophagy [88, 89] and cell reprogramming [90–92], but whether those links can also apply to β cells is yet to be clarified, especially in diabetic context. Third, the dynamic property of β cell senescence and senescence associated secretome still needs further exploration. Lastly, extensive assessments for the future of senolytic therapies against diabetes are still necessary before its application in clinical settings. In conclusion, we have summarized the recent progress in β cell senescence, through which we intend to spark more instructive discussion and perspective with regard to the mechanisms underlying β cell senescence and their links to the pathogenesis of diabetes and the development of therapeutic strategies. We believe that a comprehensive understanding of β cell senescence would provide great potential to the prevention and treatment of diabetes.
